# Lactic acidosis and hyperlactatemia associated with lamivudine accumulation and sepsis in a kidney transplant recipient—a case report and review of the literature

**DOI:** 10.1186/s12981-021-00382-8

**Published:** 2021-09-04

**Authors:** Alexa Hollinger, Nadine Cueni, Catia Marzolini, Michael Dickenmann, Emmanuelle Landmann, Manuel Battegay, Aurélien Emmanuel Martinez, Martin Siegemund, Anne Leuppi-Taegtmeyer

**Affiliations:** 1 Institute of Intensive Care, University Hospital Basel, Spitalstrasse 21, 4031 Basel, Switzerland; 2 Division of Infectious Diseases and Hospital Epidemiology, Departments of Medicine and Clinical Research, University Hospital of Basel, 4031 Basel, Switzerland; 3 Department of Transplantation Immunology and Nephrology, University Hospital Basel, Petersgraben 4, 4031 Basel, Switzerland; 4 Department of Infectious Diseases and Hospital Epidemiology, University Hospital Basel, Petersgraben 4, 4031 Basel, Switzerland; 5 Department of Clinical Pharmacology and Toxicology, University Hospital Basel, Petersgraben 4, 4031 Basel, Switzerland; 6 Medical Faculty, University of Basel, Basel, Switzerland

**Keywords:** AIDS, ART, Lactic acidosis, Lamivudine, Metabolic acidosis, NRTI

## Abstract

**Background:**

We report a case of sudden, lethal metabolic acidosis in a 70-year-old man on long-term nucleoside reverse transcriptase inhibitor (NRTI) -based antiretroviral therapy (ART) who had developed atypical necrotizing fasciitis 1 month after kidney transplantation.

**Case presentation:**

The HIV infection of the patient was treated for the last four months with an integrase strand inhibitor (dolutegravir 50 mg/d) plus a NRTI backbone including lamivudine (150 mg/d) and abacavir (600 mg/d). In this renal transplant patient we hypothesize that the co-existence of sepsis, renal failure and an accumulation of lamivudine led to the development of fatal metabolic acidosis and hyperlactatemia. Although lamivudine is only rarely associated with hyperlactatemia, there is evidence that overdose may be a risk factor for developing it. In our patient the lamivudine concentration two days after stopping and during hemodiafiltration was more than 50 times higher than therapeutic target trough concentrations. Likely reasons for this were renal impairment and concurrent treatment with trimethoprim, known to inhibit the renal elimination of lamivudine.

**Conclusions:**

NRTIs could trigger the development of hyperlactatemia in septic patients. The use of NRTI sparing regimens might be considered in the presence of this critical condition.

## Introduction

Sepsis is a major determinant of ICU admission and mortality in HIV/AIDS patients [1, 2] and may be associated with severe lactic acidosis due to tissue hypoperfusion. However, some antiretroviral drugs, notably agents belonging to the first generation of nucleoside reverse transcriptase inhibitors (NRTIs), can cause lactic acidosis and hyperlactatemia (LAHL) due to mitochondrial toxicity. NRTIs most commonly associated with LAHL (didanosine, stavudine, zidovudine) were shown to have a higher inhibitory effect on mitochondrial DNA production with a consequent reduction in mitochondrial protein synthesis leading to reduced mitochondrial function in vitro [1, 3]. Lactic acidosis has mostly been reported in the presence of one risk factor—either sepsis or NRTI treatment [1]. Little is known about the optimal management when both risk factors occur simultaneously. We present a case of LAHL with simultaneous sepsis and lamivudine accumulation and provide a summary of the available literature (identified in PubMed using the key words lactate, lamivudine and overdose), the prescribing information and society guidelines.

## Case presentation

A 70-year-old man was admitted to the intensive care unit from the nephrology outpatient clinic with sepsis and a rash on the abdomen and both forearms. Three days before ICU admission the patient was well during a routine follow-up visit at the nephrology department. He had undergone living donor kidney transplantation one month previously and the double J urethral catheter stent that was inserted after kidney transplantation was still in place. The patient was diagnosed with AIDS ten years before this when he presented with intestinal Kaposi sarcoma and candida oesophagitis. His HIV infection was suppressed (viral load < 20 copies/mL of blood, CD4 count 525 per microliter) at the time of kidney transplantation. Other comorbidities included type 2 diabetes mellitus with diabetic nephropathy diagnosed in 2014 and coronary artery disease (last percutaneous coronary intervention seven years before).

### Medication

The patient`s HIV infection was treated for the last four months with an integrase strand inhibitor (dolutegravir 50 mg/d) plus a NRTI backbone including lamivudine (150 mg/d) and abacavir (600 mg/d). Other medication prior to admission included valganciclovir (450 mg 1×/d), sulfamethoxazole/trimethoprim (800/160 mg 1×/d 3 × per week) as well as immunosuppressive therapy with tacrolimus (7 mg/d), mycophenolic acid (1440 mg/d) and prednisone (10 mg/d). Due to suspicion of a drug-induced rash, valganciclovir and sulfamethotxazole/trimethoprim were withheld on admission.

### Physical examination findings on ICU admission

A targeted mean arterial pressure of 65 mmHg was achieved by cardiovascular support with norepinephrine (4 µg/min). Other vital signs were unremarkable other than the previously known paroxysmal atrial flutter. Auscultation revealed normal heart sounds and clear lungs. Slight ankle edema was noted. The examination of the abdomen revealed multiple suffusions that were found also on both forearms upon thorough skin examination. The right forearm was oedematous and warm. The rest of his clinical examination was unremarkable.

### Diagnostic studies

Routine laboratory analyses performed three days before ICU admission revealed macrocytic normochromic anemia (hemoglobin of 104 g/L, reference range 140–180 g/L), slight thrombocytopenia (146,000, reference range 150,000–450,000 per microliter of blood), normal total leukocyte count with absolute lymphocytes of 150 per microliter of blood (reference range 900–3300 per microliter of blood), low phosphate (0.56 mmol/L, reference range 0.8–1.5 mmol/L), elevated creatinine (141 µmol/L, reference range 49–97 µmol/L), slight hypoalbuminemia (33 g/L, reference range 35–52 g/L), and slightly elevated lactate dehydrogenase (LDH, 316 U/L, reference range 135-225 U/L). Lactate and CRP were within normal limits. Therapeutic drug monitoring (TDM) of tacrolimus und mycophenolate showed concentrations of 7.9 µg/l and 4.4 mg/l (reference range at our hospital 2–4 mg/l), respectively.

Upon ICU admission, serum lactate concentration was elevated (4.8 mmol/L, reference range < 1.8 mmol/L), platelet and leucocyte counts had dropped (95,000 per microliter of blood and 1510 per microliter of blood, respectively) and CRP was markedly elevated at 452 mg/L (reference < 10 mg/L). Due to the leucopenia, mycophenolate was withheld. Within a few hours, the leukocyte count was twice as high. CD4 + count was 2 per microliter (reference range 404–1612 cells per microliter) or 8% (reference range 33–58%).

A computed tomography (CT) scan of the abdomen revealed that the transplanted kidney was imbued with fluid without disruption of drainage. Two fluid collections were seen below the transplanted kidney in the right abdomen and along the right abdominal wall. There was no radiographic evidence of fasciitis.

Blood cultures were taken at ICU admission and pre-emptive antibiotic therapy was started with piperacillin/tazobactam. Due to the skin changes antibiotic therapy was switched to imipenem and clindamycin on the same day.

### Diagnosis

We suspected necrotizing fasciitis of the abdomen and both forearms, despite the negative CT results. The patient was immediately taken to the operating room (OR) where biopsies of all areas were taken and the fasciae of the abdomen and the forearms were revised but no fasciectomy was performed.

### Clinical course

After the first operation, the patient was hemodynamically supported with low dose norepinephrine (2–4 µg/min) with lactate levels going back to normal. The patient was not intubated and respiration was unremarkable.

The second day after admission, Gram-negative rods were detected in 4/4 blood culture bottles and tissue biopsies, but not in the swabs taken from the abdomen or in the ascites sample. The patient was again taken to the OR for a second look. The fasciae still appeared inconspicuous, however fasciectomy of both forearms and partly of the abdomen was performed in order to reduce the microbial load. Slack wound closure on both sides of the lower abdomen allowed post-operative access to the fasciae, hourly lavage and eight-hourly dressing changes. Because of worsening renal function (estimated glomerular filtration rate 23 ml/min/1.7m^2^), the lamivudine dose was reduced according to the product information from 150 to 100 mg/d as per drug label recommendations (Product information 3TC, ViiV Healthcare GmbH, Münchenbuchsee, Switzerland). During the night, serum lactate concentrations rose again to 3 mmol/L but returned to normal values upon volume resuscitation.

Three days after admission to the ICU, Escherichia coli ESBL (Extended Spectrum Beta-Lactamase) was confirmed in blood cultures, abdominal swabs, biopsies, ascites and urine samples. In addition, Pseudomonas aeruginosa was detected in urine cultures. Tacrolimus was stopped due to high levels (20.5 µg/L) and progressive thrombocytopenia.

On the fourth day after admission, the double J urethral catheter stent was exchanged after the patient had become hemodynamically unstable with a maximum lactate of 5.1 mmol/L and maximum norepinephrine requirement of 12 µg/min. The patient could be stabilized after volume resuscitation. However the patient developed oligoanuric renal failure so continuous venovenous hemodiafiltration (cvvHDF) was started (pre-dilution: 1500 ml/h, dialysate 1500 ml/h, post-dilution 500 ml/h). Lactate levels remained within the normal range over the subsequent 24 h and norepinephrine could be reduced.

Five days after admission, a rapid increase in serum lactate from 1.2 to 6.3 mmol/L within three hours was observed. Despite cvvHDF, the patient developed severe metabolic acidosis (pH 6.98; bicarbonate level 8.2 mmol/l). Emergency surgical evaluation was performed, including repeat biopsies. Laparotomy and exploration of both forearms revealed no new findings on inspection, however tissue cultures again revealed growth of gram negative rods in several biopsies. Lactate levels continued to rise post-operatively. Within nine hours after the last normal value, lactate level reached 16 mmol/L. A PiCCO (Pulse Contour Cardiac Output) catheter was inserted and initially a cardiac index of 2.2 L/min/m^2^ along with a central venous oxygen saturation of 61% were measured, indicating that the cause of the hyperlactatemia was not due to severely depressed cardiac function. Norepinephrine was administered at doses as high as 50 µg/min. Argipressin (0.03 units/min), inotropes (first dobutamine, then epinephrine) and iloprost were added and a bolus of thiamine given. Despite ongoing adjustment of hemodynamic support, the patient could not be stabilized.

NRTI (lamivudine and abacavir) administration was stopped and 3 h of intermittent high-flux filter hemodialysis (IHD) were performed on ICU day five and again on ICU day six in addition to cvvHDF. Lamivudine plasma concentration measured by liquid chromatography mass spectrometry in an external laboratory [4] was 2035 ng/mL 52 h after the last 100 mg dose. The expected lamivudine steady-state through level after a 150 mg dose in patients with normal renal function is given as 38 ng/mL [5]. Dolutegravir was continued at the same dose (50 mg/d) given that it is primarily eliminated via glucuronidation. Furthermore, HIV treatment guidelines indicate that no dosage adjustment of dolutegravir is needed in case of renal impairment of hemodialysis. The patient subsequently developed liver failure with a spontaneous INR 2.2, factor V of 16% and hypoglycemia. As this occurred after the development of hyperlactatemia, liver failure was not implicated as a cause of the latter. Despite all treatment measures taken, the patient died seven days after admission. Autopsy results were consistent with a diagnosis of multiple organ failure in sepsis. Histological examination of the tissue edges of the fasciectomised areas did not reveal necrosis or inflammation.

The patient’s sister gave written consent for publication of this case. The case was also reported anonymously to the pharmacovigilance department of the Swiss national drug authority (Swissmedic) according to local laws and regulations.

## Discussion

In this renal transplant patient with HIV, the presence of several co-existing factors likely led to the development of lactic acidosis and hyperlactatemia. The potential influence of tacrolimus (and other renally active drugs) and their impact on acute kidney injury besides 3TC should also be taken into consideration: Tacrolimus shows dose-dependent nephrotoxicity and the high levels measured three days after admission are likely to have contributed to the worsening renal function. Tacrolimus was stopped due to high levels and progressive thrombocytopenia only three days after ICU admission when the patient was diagnosed with sepsis.

Lactic acidosis is associated with a poor prognosis in a variety of conditions such as sepsis or liver failure [6, 7] and is a known adverse drug reaction of first generation NRTIs (e.g., didanosine, stavudine and zidovudine) [8]. Newer NRTIs such as lamivudine, abacavir and emtricitabine have only rarely been associated with lactic acidosis [8]. A case report from 2018, however, describes a patient who developed reversible lactic acidosis and hyperlactatemia (9.2 mmol/l) in the setting of drug overdose after having ingested 9 g of lamivudine and 18 g of abacavir [9].

Plasma lamivudine concentration 52 h after stopping the drug were elevated more than 50-fold compared to the reference concentration. By comparison, mean steady-state trough lamivudine concentrations after daily administration of 150 mg in healthy subjects with normal renal function was 38 ng/ml [5]. Reasons for the lamivudine accumulation in our patient were likely the combination of renal impairment (lamivudine is predominantly excreted renally via cation transporters) and a pharmacokinetic interaction with trimethoprim (a cation transporter inhibitor) [10, 11]. Although the co-administration of 160 mg trimethoprim daily and lamivudine causes a 40% increase in lamivudine exposure, no dose-reduction is recommended in patients with normal renal function (Product information 3TC, ViiV Healthcare GmbH, Münchenbuchsee, Switzerland).

The last lamivudine dose was administered while the patient was on cvvHDF and plasma lamivudine concentration sampling was performed after two additional dialysis periods. Nevertheless, lamivudine concentrations were very high indicating that lamivudine was not efficiently cleared by haemodiafiltration or dialysis. This is likely due to both lamivudine`s large volume of distribution (1.3 L/kg body weight [12]) and its active tubular secretion via cation transporters [11]. The Swiss product information mentions that lamivudine is dialyzable and dialysis could be considered in the treatment of overdose (Product information 3TC, ViiV Healthcare GmbH, Münchenbuchsee, Switzerland). A study in nine patients undergoing intermittent hemodialysis, however, found that maximum plasma concentrations and exposure were not significantly affected by dialysis and that based on pharmacokinetic modelling, a lamivudine dose of 25 mg provided a comparable exposure to 150 mg in patients with normal renal function [13]. Dosing guidelines for patients undergoing dialysis or continuous renal replacement therapy given by the European AIDS Clinical Society Guidelines are 25–50 mg lamivudine daily (The European AIDS Clinical Society Guidelines available at https://www.eacsociety.org/guidelines/eacs-guidelines/eacs-guidelines.html, last accessed September 2020).

TDM of lamivudine in plasma is not routinely performed as efficacy relates to intracellular concentrations. However, monitoring of plasma concentrations (where available) could be useful in patients with fluctuating renal function to detect lamivudine accumulation early on and allow appropriate action to be taken in a timely fashion.

LAHL is a rare, serious side effect of NRTI treatment today and is considered to arise from NRTI-induced mitochondrial toxicity [6]. This is explained by structural similarities between human mitochondrial DNA polymerase and HIV-reverse transcriptase representing the NRTI target [6]. Although clinically not clearly described [3], a dose-dependence seems plausible on the basis of in vitro studies [14] and the recently described overdose case [9].

In our opinion, our patient had co-existing causes of LAHL, namely; severe sepsis (triggered by bacterial infection in the setting of post-transplant immunosuppressive therapy) and a high lamivudine exposure. Furthermore acute kidney injury as a consequence of the gram-negative sepsis might also be the reason for 3TC accumulation. As such, increased immunosuppressant levels, sepsis, acute renal failure, lamivudine accumulation and LAHL could be considered to be a cascade of related events. The Naranjo Adverse Drug Reaction probability score was five indicating a probable ADR (score 5–8 are judged by this scale as probable while scores ≥ 9 are judged as definite) [15].

## Conclusions

We describe a case of LAHL in an HIV-patient under immunosuppression and NRTI-treatment who developed sepsis, impaired renal graft function and lamivudine accumulation. It is currently not possible to elucidate the individual contributions of sepsis and NRTI accumulation to the development of LAHL in such cases, in part because point of care plasma lamivudine concentration testing is not available. This poses diagnostic and therapeutic dilemmas to the treating healthcare professionals. Whether routine TDM of lamivudine after transplantation, or temporary withdrawal or replacement of NRTI treatment by another antiretroviral at the onset of sepsis could have led to an improved outcome is not clear, however these options could be considered in future similar cases.

## Data Availability

The datasets used and analysed during the current study are available from the corresponding author upon request. The clinical data are stored electronically in the intensive care clinical information system software (MetaVision, iMDsoft^®^) provided in the intensive care unit of the University Hospital Basel.
